# Lévy Flight Model of Gaze Trajectories to Assist in ADHD Diagnoses

**DOI:** 10.3390/e26050392

**Published:** 2024-04-30

**Authors:** Christos Papanikolaou, Akriti Sharma, Pedro G. Lind, Pedro Lencastre

**Affiliations:** 1 Department of Computer Science, Oslo Metropolitan University, N-0130 Oslo, Norway; c.papanikolaou@windowslive.com (C.P.); akritish@oslomet.no (A.S.); pedrolin@oslomet.no (P.G.L.); 2 OsloMet Artificial Intelligence Lab, Pilestredet 52, N-0166 Oslo, Norway; 3 NordSTAR—Nordic Center for Sustainable and Trustworthy AI Research, Pilestredet 52, N-0166 Oslo, Norway; 4 Simula Research Laboratory, Numerical Analysis and Scientific Computing, N-0164 Oslo, Norway

**Keywords:** ADHD diagnosis, eye-tracking data, gaze trajectories, Lévy flights, Lévy foraging hypothesis

## Abstract

The precise mathematical description of gaze patterns remains a topic of ongoing debate, impacting the practical analysis of eye-tracking data. In this context, we present evidence supporting the appropriateness of a Lévy flight description for eye-gaze trajectories, emphasizing its beneficial scale-invariant properties. Our study focuses on utilizing these properties to aid in diagnosing Attention-Deficit and Hyperactivity Disorder (ADHD) in children, in conjunction with standard cognitive tests. Using this method, we found that the distribution of the characteristic exponent of Lévy flights statistically is different in children with ADHD. Furthermore, we observed that these children deviate from a strategy that is considered optimal for searching processes, in contrast to non-ADHD children. We focused on the case where both eye-tracking data and data from a cognitive test are present and show that the study of gaze patterns in children with ADHD can help in identifying this condition. Since eye-tracking data can be gathered during cognitive tests without needing extra time-consuming specific tasks, we argue that it is in a prime position to provide assistance in the arduous task of diagnosing ADHD.

## 1. Introduction

The study of time-series is central to mathematics and physics with an extensive range of interdisciplinary applications. A class of time-series that has gathered increased interest in recent years is the one with rare extreme events, an example of which is the recording of human eye-movements while looking at a screen, i.e., eye-tracking. It is currently an open question what is the most suitable mathematical equation describing gaze trajectories. There are two schools of thought: one that claims that gaze trajectories are two alternating subprocesses and another that claims that gaze trajectories are described by a single, scale-independent process [1].

In the first description, the two alternating types of eye-movements are *fixations*, during which an individual’s gaze is centered around one specific point with very little deviation and, presumably, visual information is being extracted, and *saccades*, fast re-locations of the gaze from one point to another [2]. In the second mathematical model, it is posited that the visual and oculomotor systems may conform to the Lévy flight model [3]. This has been empirically supported by the work presented in Ref. [4], with the emergence of superdiffusive dynamics arising in this context [5]. In fact, the superdiffusive nature of gaze trajectories has been presented over two decades ago [6]. In that study, the authors proposed that visual scan-paths generated under natural circumstances bear similarities to Lévy flights, indicating a power-law dependency in their magnitude distribution, a phenomenon that occurs even in tasks with pre-planned positions for the visual targets [7]. In the context of visual scan-paths, this indicates that a viewer’s attention may occasionally make extensive shifts across the scene, intermixed with smaller, local movements. It has also been shown that Lévy flights reproduce human gaze patterns well over a saliency map [8].

This is similar to the foraging subclass of stochastic processes, i.e., time-series where an agent is searching for food, water, or, in our particular case, visual information. Indeed, it is speculated that Lévy flights describe foraging strategies observed in nature in all its contexts [9]. These strategies are manifest in the movement of bacteria [10,11], the search for fish by albatrosses [12], or the foraging for food by sharks in the ocean [13]. This pattern is also present in several human tasks, such as the exploration of the Walt Disney Resort by children [14], the exploration of virtual environments [15], and was even proposed as a mathematical model for the way humans search for memories [16] or the response time in decision making based on the accumulation of evidence [17,18,19]. Using this framework, eye-tracking experiments found the characteristic Lévy exponent (α) to be approximately α=0.9 (or α=1.9 using a different convention than the one we are using here) [20], regardless of the level of difficulty of the visual task.

While it is still an open question if eye-gaze trajectories follow Lévy-like processes or others, such as intermittent processes [21], this finding supports the notion that the Lévy flight model can be applied to visual scan-paths, and it emphasizes the potential relevance of the scaling exponent α in characterizing eye movements. The question of universality is not new, and it has been argued that an optimal α for search strategies is α=1 [22].

Besides the theoretical question of the best mathematical description of gaze trajectories, eye-tracking data has been gaining popularity due to its wide range of applications in health sciences. It can be used to determine an individual’s age, gender, ethnicity [23], personality traits such as extroversion, curiosity, neuroticism, openness, agreeableness [24], Intelligence Quotient (IQ) [25], as well as helping detect conditions such as Alzheimer’s [26], autism [27], or dyslexia [28,29].

Due to its diagnostic challenges, one of the conditions where eye-tracking might have the most impact is Attention-Deficit/Hyperactivity Disorder (ADHD). It has been suggested that around 20% of ADHD diagnoses could be false positives [30] with an unknown expected percentage of children classified as false negatives. Indeed, significant differences in gaze dynamics were found in people with ADHD, namely regarding fixation durations in reading tasks [31] and saccade abnormalities during working memory tasks [32].

While the condition is not curable, an early diagnosis is important to mitigate its impact, provide adequate medication, and teach patients coping skills. This is increasingly important when taking into account that it is the most prevailing neurodevelopmental disorder among children and adolescents, impacting academic performance, overall well-being, and social interactions in children [33]. When unmitigated, the impact can be felt even in adulthood, with affected individuals having shorter and more discordant romantic relationships [34].

In the important task of assisting with ADHD diagnoses, eye-tracking has been applied in reading tasks [31], during memory performance tests [35], or while playing in virtual reality [36]. We intend to add to this body of literature by justifying and applying a Lévy flight-inspired methodology. These methods also have the advantage of being scale invariant and, thus, in a time when phone cameras can start tracking eye-movements [37], the robustness of this approach to different scales being employed is a definite advantage.

This paper is structured as follows: in Section 2, the methods applied, and the data used are introduced; in Section 3, the results of our analysis are presented. Finally, in Section 4, we present the conclusions of the paper.

## 2. Data and Methods

### 2.1. Data: Eye-Gaze Trajectories and ADHD Cognitive Test

The dataset we used in this study originates from an experiment detailed in Ref. [38], focusing on visuospatial working memory and involving participants both with and without ADHD. ADHD in children was identified by a trained child neurologist following the DSM-IV criteria [39] with Te Conner’s Abbreviated Parent–Teacher Questionnaire [40] being used as an additional metric to count ADHD symptoms. Comorbidities were evaluated using a general medical opinion, parent interview, and the M.I.N.I. Kid test [40]. Further details can be found in Ref. [41]. The experiment included 50 participants engaged in a Sternberg-type delayed visuospatial working memory task, adapted from Ref. [38]. From these 50 participants, 47 had complete data records, with 21 children diagnosed with ADHD and 26 without.

This working memory task showed one or two dots on the screen, with dots positioned in one of sixteen locations within a 4 × 4 grid. The goal of the participants was to memorize the position of the dots, while some distractors (images or videos) were shown between the time when the participants first saw the dots and the time when they were finally evaluated on memorizing the dots’ position.

The assumption nested in this experiment is that children with ADHD are more prone to being distracted and, thus, will fail this test more often. This assumption also arises in the studies from Refs. [42,43], which used the WISC-III cognitive test and found that the ADHD group scored significantly lower on Freedom from Distraction (FD), which, in the context of the WISC-III cognitive test, is composed of the Digit Span and Arithmetic subtests. Results of the WISC-III cognitive test are also provided in this dataset.

Other parameters that might affect a child’s performance in the memory test were controlled for, namely, age (10.71±0.54 years for the ADHD group and 11.58±0.50 years for non-ADHD) and IQ (100±11.6 for ADHD and 105±7.3 for non-ADHD). Eye movements and pupil diameter were recorded for the duration of the experiment using Eyelink 1000, a powerful eye-tracker with a 1 kHz sampling frequency. Fixation and saccade labeling of data points were not provided. To create such classification, a velocity-based Hidden Markov Model identification algorithm was implemented, the details of which can be found alongside the code of our analysis [44].

### 2.2. Lévy Flights and Superdiffusion

A Lévy flight is a type of random walk named after the French mathematician Paul Lévy, which has seen a wide range of applications since the 1980s [45]. As a random walk, it describes a series of positions across time via the equation

(1) $$\vec{R}\left(t\right)=\vec{R}\left(0\right)+\sum_{i=1}^{N(t)}\Delta {\vec{r}}_{i}\;,$$

where $\vec{R}\left(t\right)$ is the position of the particle at time *t*, $\vec{R}\left(0\right)$ the initial position, $\Delta {\vec{r}}_{i}$ the position increments or steps, and N(t) is the number of steps taken up to time *t*.

The distribution of the increments, $\Delta {\vec{r}}_{i}$, is what distinguishes a Lévy flight from other random walks such as Brownian motion [46]. Unlike the latter, where $\Delta {\vec{r}}_{i}$ are normally distributed, in a Lévy flight, the steps follow a heavy-tailed distribution characterized by its power-law tail.

The right-sided tail of the probability density function (PDF) of a Lévy flight is given by

(2) $$p(|\Delta \vec{r}\left|\right)\propto \frac{1}{|\Delta \vec{r}{|}^{\alpha +1}}\;,$$

where the coefficient 0<α<2 determines the heaviness of the tails [47]. When α>2, the process converges to a Gaussian random walk [48] by the central limit theorem, while for values α≤0, the process does not yield a normalized probability distribution. For 0<α≤1, the distribution has no convergent mean and variance, and for 1<α≤2, the distribution has a finite mean but divergent variance [21]. This is why this type of distribution escapes the range of applications of the central limit theorem (which requires a finite mean and variance) and does not converge to a Gaussian random walk when the steps are measured over large time-windows, $\tau =N\Delta t_{s}$, where *N* is an integer, and $\Delta t_{s}$ is the interval of time at which the data is sampled, which, in our case, is $\Delta t_{s}=10^{-3}$.

The fact that, asymptotically, Lévy flights lead to a different behavior than the Wiener process is well documented in the literature, with this behavior being known as superdiffusivity [5], a phenomenon characterized by a higher-than-normal rate of spread or dispersion compared to classical diffusion. In a superdiffusive process, the mean-squared displacement (MSD) of a particle grows faster than linearly with time. The MSD is defined as

(3) $$\mathrm{MSD}\left(\tau \right)=\langle |\vec{R}\left(\tau \right)-\vec{R}\left(0\right){|}^{2}\rangle \;.$$

For classical diffusion, the MSD grows linearly with time (MSD(τ)∝τ), but, in superdiffusion, the growth is faster than linear with

(4) $$\mathrm{MSD}\left(\tau \right)\propto \tau^{\gamma }\;,$$

with, in the context of Lévy flights,

(5) $$\gamma =\frac{4}{2+\alpha }\;.$$

Multifractal detrended fluctuation analysis (MFDFA) is a technique that can be used to estimate the parameter γ. It is an extension of the detrended fluctuation analysis (DFA) method, allowing for the assessment of the multifractal nature of complex systems. The details of the algorithm can be found in Ref. [49].

### 2.3. Machine Learning Classification Methods

In order to classify individuals as belonging to the ADHD category or not, based on parameters such as age, test performance, or features of their gaze trajectories, we employed three different machine learning methods for this type of classification task.

#### 2.3.1. Logistic Regression

Logistic regression is one of the most simple binary classification methods. In it, the logistic function, also known as the sigmoid function, is used to map the output of a linear combination of features to a value between 0 and 1.

The logistic function is defined as

(6) $$\sigma \left(z\right)=\frac{1}{1+e^{-z}}\;,$$

where *z* is the linear combination of input features and model parameters

(7) $$z=\beta_{0}+\beta_{1}x_{1}+\beta_{2}x_{2}+\ldots +\beta_{n}x_{n}\;.$$

In our case, the x variables represent metrics such as age, IQ, or eye-tracking-derived metrics, which we will use to distinguish between children with ADHD and those without the condition.

Conversely, the β-coefficients associated with x are estimated via a maximum likelihood estimation and each coefficient $\beta_{i}$ can be interpreted as the weight of the variable $x_{i}$ to the model, with $\beta_{0}$ being the intercept of the model.

After estimating the β-coefficients, one can calculate the probability of an instance characterized by a variable array *x* belonging to the positive class:

(8) P(Y=1)=σ(z),

where *Y* is the binary target variable, where 1 corresponds to the case of a child with ADHD and 0 otherwise.

The logistic regression model predicts the class label by thresholding the predicted probability as follows:

(9) $$\hat{y}=\left\{\begin{array}{cc} 1 & \mathrm{if}\;P(Y=1)\geq 0.5\\ 0 & \mathrm{if}\;P(Y=1)<0.5\;. \end{array}\right.$$

#### 2.3.2. Support Vector Machines

Support Vector Machines (SVMs) are another class of supervised algorithms used for classification tasks. In this context, this algorithm has the goal of finding the hyperplane that best separates the data into different classes. In our case, that creates two mutually exclusive regions where, given the value of the feature vector x, an instance is assigned a value Y=0 or Y=1.

In the most simple case, when a hyperplane exists that perfectly separates between classes (ADHD and non-ADHD, in our case), the hyperplane defined by the SVM algorithm has the form

(10) f(x)=w·x+b,

where, similarly to the β-coefficients in the logistic regression, the vector w represent the weight vector and *b* represents the bias term (similarly to $\beta_{0}$ in the logistic regression).

In that case, the classification decision is made based on the sign of f(x):

(11) $$\hat{y}=\left\{\begin{array}{cc} 1 & \mathrm{if}\;f(x)\geq 0\;,\\ 0 & \mathrm{if}\;f(x)<0\;. \end{array}\right.$$

In most practical applications, it is not possible to find a hyperplane that perfectly separates between classes and some overlap exists, even after finding an appropriate hyperplane. In this case, SVMs can use kernel functions to map the input features into a higher-dimensional space. The decision function becomes

(12) $$f\left(x\right)=\sum_{i=1}^{N}\alpha_{i}y_{i}K\left(x_{i},x\right)+b\;,$$

where $K(x_{i},x)$ is a kernel function and $\alpha_{i}$ are the Lagrange multipliers.

Common kernels include the linear kernel $K\left(x_{i},x\right)=x_{i}\cdot x$, the polynomial kernel $K\left(x_{i},x\right)={\left(x_{i}\cdot x+c\right)}^{d}$, and the radial basis function kernel $K\left(x_{i},x\right)=\exp\left(-\frac{∥x_{i}{-x∥}^{2}}{2s^{2}}\right)$, with *s* being a width parameter. Here, we used a linear kernel in our analysis. To avoid overfitting, in the case of SVM and logistic regression, we used the Ridge regularization with the strength parameter chosen via cross-validation.

#### 2.3.3. Decision Trees and Random Forests

A decision tree is a non-linear, non-parametric model used for both classification and regression tasks. A decision tree recursively partitions the feature space (where *x* lies) to create homogeneous subsets until a stopping criterion is met. The decision at each node is typically based on optimizing impurity measures such as Gini impurity or entropy.

Mathematically, a vector x encapsulates the relevant metrics for a classification task and a series of corresponding labels y. A decision tree can be represented by a function f(x) that maps feature vectors to predicted labels:

(13) $$f\left(x\right)=\sum_{i=1}^{N}w_{i}\cdot I\left(x\in R_{i}\right)\;,$$

where *N* is the number of terminal nodes, $w_{i}$ the predicted value at terminal node *i*, $R_{i}$ the region corresponding to terminal node *i*, and I(·) is the indicator function. Here, we chose *N* via cross-validation and found 8 to the optimal number for this quantity.

A Random Forest is a method that groups multiple decision trees and combines their predictions. Random Forests improve generalization and reduce overfitting compared to individual decision trees. Each tree is trained on a subset of the data and may use a random subset of features at each split. The final prediction is a majority vote of individual tree predictions.

### 2.4. Cross-Validation Strategies

Given the limited number of participants, we could not use the common approach of fitting the parameters of the model in 80% of the data and testing its accuracy in the remaining 20%. Instead, we used different cross-validation (CV) strategies.

In the first CV strategy, we employed an 80%−20% split, choosing a different combination of participants each time to fit the parameters and test the accuracy of the model, in a process that is known as the Stratified K-Fold (SKF) CV (k=5 in this case). The final accuracy reported an average of 5000 80%−20% combinations.

In the second type of CV, we applied a Leave One Out cross-validation (LOOC), where we fit the parameters of a model with all the data except for 1 entry, which was then left to be tested. In our case, with 47 participants in total, this was done 47 times, each time fitting the parameters of the model on 46 entries and testing the model’s accuracy on 1 participant. Again, the reported accuracy was the average over all 47 realizations.

## 3. Results: Lévy Flight Exponents to Identify ADHD Children

**Figure 1:**
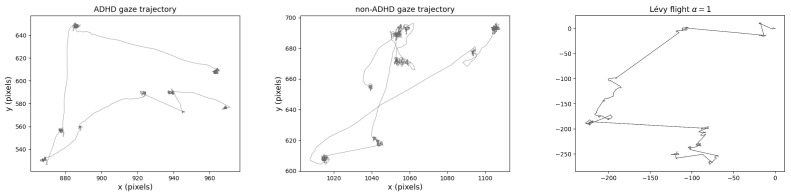
Example of one gaze trajectory for ADHD (**left**) and non-ADHD (**middle**) children, composed of 1500 data points, corresponding to 1.5 seconds of visual engagement. On the (**right**), we show a simulated Lévy flight with α=1.

From here onwards, we will analyze the data from the one-dot memorandum task. In this task, participants saw one dot in a 4 × 4 grid and had to memorize its position for 1.5 s, a period of time during which they were shown pictures and videos that served as distractors. Since these distractors varied from trial to trial, for consistency reasons, the eye-tracking data that we analyzed focused solely on the gaze patterns of participants while looking at the 4 × 4 grid with one dot in it (Figure 1).

### 3.1. Eye-Gaze Dynamics and the Lévy Flight Exponent

**Figure 2:**
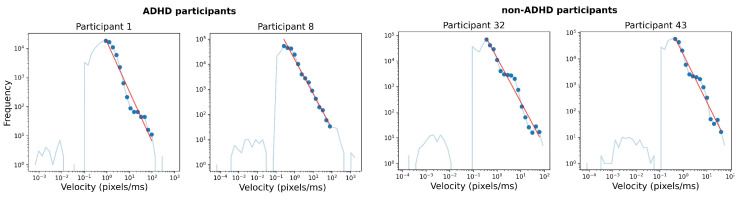
The probability density function of the increment norm for different experiment participants. The power-law decay of the PDF is marked with a red line.

While the Lévy flight framework diverges from the usual picture of two alternating processes, one slow (called *fixation* in the oculomotor field), one fast (called *saccades*), in our case, we can see that not only is it adequate, but actually preferable. In Figure 2, we observe two relevant features in the PDF of the distribution of the increment’s norm $∥\Delta \vec{r}∥$: firstly, we observe a power-law decay that is integral to the PDF and not just a small part of the tail of the pdf and, secondly, we do not observe a bi-modal velocity PDF that is often used as a justification for the assumption of two separate subprocesses of fixations and saccades. To estimate α from the PDF, we firstly identified the mode *M* of the distribution. From that point, we fixed the constant factor *k* and calculated the linear regression in the log–log PDF plot in the interval [M,M×k]. The quantity *k* is chosen with the goal of maximizing the average $R^{2}$ across all participants and, in our case, it was chosen as 5.9.

**Figure 3:**
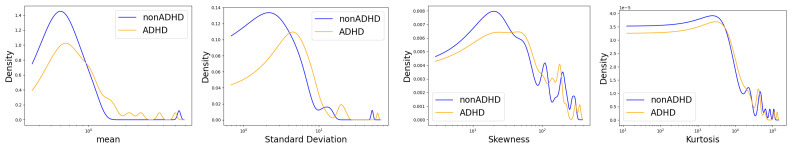
The probability density for the distribution of the first statistical moments of $∥\Delta \vec{r}∥$ across participants: mean (**top left**), standard deviation (**top right**), skewness (**bottom left**), and the kurtosis (**bottom right**).

We also see, in Figure 3, the values of the mean, standard deviation, skewness, and kurtosis of $∥\Delta \vec{r}∥$. We observe that they are quite disparate across experimental participants, often with variations of two orders of magnitude. That is not surprising, if we consider that these quantities are divergent in a Lévy flight model, with their values being extremely sensitive to small parameter variations. One further advantage of looking at the Lévy exponent α (or for the scaling exponent γ) is that this approach is scale invariant.

**Figure 4:**
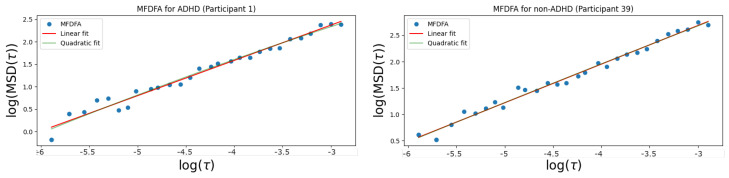
Representation of the MSD as a function of the time window τ. The line in green represents the best quadratic fit to the data points, while the red one represents the best linear fit. We observe that, on a log–log plot, the empirically estimated points fall along a straight line, which corroborates the relation $\mathrm{MSD}\left(\tau \right)\propto \tau^{\gamma }$.

**Figure 5:**
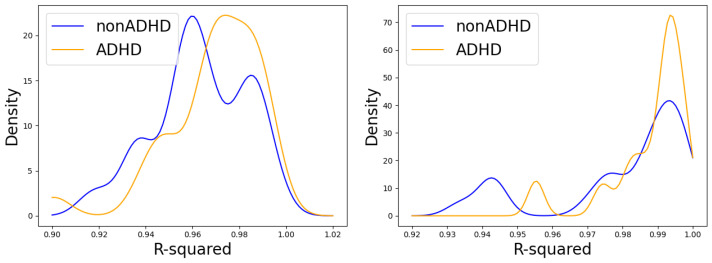
The probability density of the R-square for the straight line fits on the log–log plot of the PDF decay, by virtue of which α is estimated (**left**). On the (**right**) is shown the coefficient of determination corresponding to the straight line fit on the MFDFA.

We observe as well, in Figure 4, that the relation $\mathrm{MSD}\left(\tau \right)\propto \tau^{\gamma }$ holds to a significant degree. Furthermore, in Figure 5, we computed the coefficient of determination (r-square). With values of r-square over 0.9, we see that both the estimation of α via the PDF and the estimation using the relation $\mathrm{MSD}\left(\tau \right)\propto \tau^{\gamma }$ corroborate the Lévy flight assumption as a valid one. The MFDFA method is often considered the most robust algorithm to estimate the parameter α and, in Figure 5, we see that it generates better quality fits. It is thus this method that we use in what follows.

### 3.2. Classifying ADHD without Eye-Tracking

Before investigating the impact of including a Lévy description of gaze movements in our classification model, we created a benchmark model using other metrics solely dependent on non-eye-tracking related metrics. These are the age of the children, the specific metrics from their WISC test, and their test performance. The accuracy of this model will subsequently be compared with the model, including eye-tracking metrics.

To create the benchmark model, we used logistic regression with the following variables: age, full-scale IQ, arithmetic and digit span subtests of the WISC-III cognitive test, and the one-dot memory test performance. While we had fifty test participants, three of them had incomplete data, leaving us with forty-seven test subjects.

The results are presented in Table 1. There, we see that, in either case of CV strategies, the logistic regression is the best-performing method, with an accuracy of 72.3%, regardless of the type of CV. Thus, we will use the LOOC from now onwards, since we can more easily cover all possible CV combinations. The model results can also be seen in Table 2 (left), with other metrics such as a precision of 72.4%, specificity of 61.9%, and recall 80.7%. The IQ variable was not included in the analysis, as using it led to overfitting and a decrease in CV accuracy.

### 3.3. Classifying ADHD with Eye-Tracking

**Figure 6:**
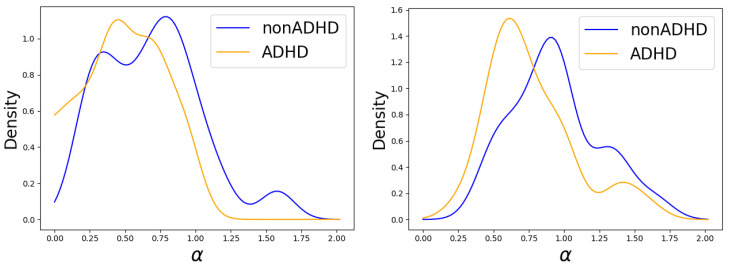
Lévy exponent α distributions for ADHD and normally developed children are calculated via the increment’s PDF (**left**) and via the MFDFA method (**right**). The average standard error for the α estimation was 0.08 (maximum of 0.22) for the PDF method and around 0.03 (maximum of 0.08) for the MFDFA method. A Mann–Whitney U statistical test confirms that the difference in medians is significant, with a *p*-value of around p=0.037 (PDF estimation) and p=0.012 (MFDFA estimation).

When we calculated the value of the Lévy exponent α, we found that there was a discrepancy between children with and without ADHD. We see in Figure 6 that the median of α for children without ADHD is around α=1, while, for children with ADHD, it is centered between 0.6 and 0.8. According to the Mann–Whitney U test, this difference is significant with a *p*-value of around 0.01.

While there was a difference in the median of the distribution of α, this parameter alone did not yield better accuracy than the benchmark model (around 60%). However, when incorporated with the other variables, it did increase the accuracy of the logistic regression from 72.4% to 76.5% (see Table 2 on the right). The accuracy increase for all the other models is reported in Table 1.

The increase in accuracy was reflected in the other metrics, namely, the precision (from 72.4% to 77.7%) and specificity (from 61.9% to 71.5%). Even though this increase was not very substantial, we must take into account that we had a limited amount of data. When this limitation takes place, typically, adding more variables can lead to overfitting, which can decrease the overall accuracy. In fact, when using cross-validation and recursive feature elimination, we excluded the participants’ IQ from our model, as it worsened the accuracy of the model. It is expected that, if more data were available, the accuracy increase would also be higher, and it is unclear if IQ would be excluded from the classification model in that circumstance.

Children with ADHD typically had a lower α coefficient, indicating that they were more prone to having large, extreme relocations and exploring a larger area of the screen image. In this context, gaze dynamics mimicked some of the body language of children with ADHD, which often seems uneasy with a tendency for extra movements when engaged in tasks that require concentration. Children without ADHD presented a value of α close to 1. This value is quite significant in the literature, as a conjecture exists stating that α=1 reflects the optimal strategy for searching processes [22,50,51,52,53]. It is the same value that was found in other contexts, such as memory retrieval [16], and is close to the values of the exponents found in other eye-tracking studies [20,54]

These results were replicated in another dataset [55] with ADHD and typically developed children, and we found a similar α=1 exponent for typically developed children. Furthermore, while a difference in α exponents was not found when analyzing $|\Delta \vec{r}|$, a significant difference in the α distribution’s mean (p=0.015) and median (p=0.015) was found for the horizontal increments $|\Delta \vec{x}|$ and for the median (p=0.015) of the vertical increments $|\Delta \vec{y}|$. In this dataset, without additional cognitive tests, using only α as a variable in a logistic regression model resulted in an accuracy of 66.9%. All the data and code of our analysis, including the replication of our results in a different dataset, can be found in Figshare [44].

## 4. Discussion and Conclusions

In this work, we argued that Lévy flights are good mathematical models of gaze dynamics and that their characteristic exponent α is distributed differently between ADHD and non-ADHD children. This is a natural expectation, given that this is a defining quality of gaze patterns, which, in turn, reflect attention patterns. Our findings show that having a typically smaller α exponent, ADHD children explore more remote areas of images, which might be a consequence of their attention deficit. This contrasts with the case of autism, another neurodevelopmental condition, where it was found that children with this condition have gaze trajectories with a larger α Ref. [54].

We observe that the Lévy flight description of gaze dynamics can improve the classification accuracy that is obtained when using a cognitive test. While the improvement is not very substantial, this also stems from the fact that we have a limited amount of data, and, when that is the case, adding extra variables to an already multi-variable model can lead to overfitting or to reduced marginal accuracy gains. In particular, using cross-validation, we eliminated the participants’ IQ as a variable of our classification model, which might prove to be useful in an expanded dataset. Moreover, the classification accuracy that we obtained by analyzing gaze dynamics is in line with the one obtained by performing the pupil diameter analysis [56], which is the quantity of focus in the dataset description [38].

The increase in accuracy when using the Lévy model stands in contrast to the case when one uses traditional eye-tracking metrics, namely the duration of saccade and fixation and their respective average velocities. When average saccadic velocity was added to the logistic regression model, classification accuracy remained stable (72.4%). However, accuracy decreased to 68.0% when we included average fixation velocity or fixation and saccade durations in the logistic regression model. Other studies have used metrics based on accurate gaze shifts and gaze duration applied to other datasets, namely autism [54]. In that study, the authors reported an accuracy up to 76% using standard eye-tracking metrics. For this other dataset, we obtained, with our method, an accuracy of 77.2%. More recently, we also analyzed the same dataset of autistic children [57] to investigate the ability to detect autism using a method, not based on eye-gaze velocity, but on a sort of visited area of the eye-gaze, measuring how “exploratory” a specific eye-gaze is. There, we obtained an accuracy of 93%, even when using shorter trajectories (less data). Other studies have analyzed reading tasks to investigate dyslexia [28,29], with similar standard metrics; the authors aimed to characterize the eye movements of children with dyslexia.

All in all, our method, based on the exponent of the Lévy flight model, presented an overall good performance, while other traditional metrics also showed a comparable level of performance when applied in other datasets; the Lévy flight model can be regarded as an additional method to assist in diagnosing ADHD. An interesting next step in the context of stochastic statistical physics, which can be explored for an extension of the methodology proposed here, deals with the so-called detailed fluctuation theorem. This theorem expresses the balance between entropy–consumption (positive variations of entropy, ΔS) and entropy–production (negative variations of entropy) of particular stochastic trajectories, and states that the quotient between the probability of each one, i.e., p(ΔS)/p(−ΔS), is proportional to exp(ΔS) [58]. Therefore, by computing the entropy fluctuations of eye-gaze trajectories, one can derive the distribution of entropy fluctuations for both groups of children, with and without diagnosed ADHD, which might further improve the accuracy of our method.

Our approach also has the advantage of being scale-independent. This means that it is not as sensitive to particular experimental parameters, such as the eye-tracker’s native frequency or the screen distance, as other approaches. Consequently, our approach could be used in eye-trackers with different characteristics and operated outside strict laboratory conditions. This is of particular importance for assisting in the diagnosis of ADHD in children, who typically present challenges for laboratory experimental setups.

## Figures and Tables

**Table 1:** Accuracy values for different models using SKF and LOOC CV strategies. The two top rows show the accuracy benchmark for models without including α as a model variable, while the bottom row shows the LOOC accuracy of the models including α.

α in Model	Type of CV	Logistic	SVC	Decision	Random
Variables		Regression		Tree	**Forest**
Excluding α	SKF (k = 5)	0.724	0.680	0.611	0.680
Excluding α	LOOC	0.724	0.724	0.680	0.724
Including α	LOOC	0.765	0.765	0.724	0.744

**Table 2:** On the left, the confusion matrix of the logistic regression benchmark model with an accuracy of 72.3%, precision of 72.4%, specificity of 61.9%, and recall 80.7%. On the right, we have the confusion matrix, including the benchmark parameters as well as the Lévy exponent, yielding an accuracy of 76.5%, precision of 77.7%, specificity of 71.5%, and recall of 80.7%.

	Predicted Label				
**True label**		**ADHD**	**Non-ADHD**	**ADHD**	**Non-ADHD**
	**ADHD**	13	8	15	6
	**non-ADHD**	5	21	5	21

## Data Availability

This study used open available data as stated in the manuscript (Section 2).
